# Unveiling the Uncommon: A Rare IgE-Mediated Presentation of Multiple Drug Hypersensitivity Syndrome

**DOI:** 10.7759/cureus.100855

**Published:** 2026-01-05

**Authors:** Ana Vitória Soares, Diogo Mota, Daniela Brandão Abreu, Eunice Dias-Castro, Leonor Carneiro-Leão

**Affiliations:** 1 Allergy and Clinical Immunology Department, Centro Hospitalar Universitário São João, Porto, PRT; 2 Basic and Clinical Immunology Department/Pathology Department, Faculty of Medicine, University of Porto, Porto, PRT; 3 Public Health, Forensic Sciences, and Medical Education Department, Faculty of Medicine, University of Porto, Porto, PRT

**Keywords:** ige-mediated, mrgprx2 receptor, multiple drug hypersensitivity syndrome, nsaids hypersensitivity, quinolones

## Abstract

Multiple drug hypersensitivity syndrome (MDHS) is characterized by hypersensitivity reactions to two or more unrelated drugs. Although it is commonly associated with delayed reactions, we report a rare case of immediate IgE-mediated MDHS in a 30-year-old female who reacted to levofloxacin and ibuprofen. Both reactions were confirmed through drug challenges, while acetylsalicylic acid was tolerated, excluding COX-1 cross-reactivity. This case highlights the importance of recognizing IgE-mediated MDHS and performing thorough allergy diagnostic workups to ensure accurate management and patient safety.

## Introduction

Multiple drug hypersensitivity syndrome (MDHS) was first described in 1989 and is a unique clinical entity characterized by drug hypersensitivity reactions (DHSR) to two or more different drugs that are chemically and structurally unrelated. This term should be reserved only for well-documented cases in which drug involvement has been demonstrated through allergy diagnostic workup [1]. Confusion with multiple drug intolerance syndrome (MDIS) and cross reactivity, from which it should be clearly distinguished, makes it challenging to determine the actual prevalence of patients with MDHS. However, retrospective analyses reported a prevalence of 0.56% [2].

MDHS is frequently documented in non-immediate severe reactions, such as drug reaction with eosinophilia and systemic symptoms (DRESS), where it can complicate up to 18% of cases [3]. The pathophysiology of MDHS remains unclear. A study found that, unlike monoallergic patients with maculopapular exanthema (MPE), MDHS patients retained activated T cells (CD4+, CD25dim, CD38+, PD1+) for months to years after initial symptoms resolved. These T cells resemble those seen in graft-versus-host disease, suggesting that MDHS may involve a chronic, graft-versus-host-like reaction stemming from an acute drug hypersensitivity (DHS), leading to ongoing immune stimulation [4].

MDHS is classified into simultaneous, sequential, and distant forms [1]. The simultaneous form is considered when sensitizations to more than one drug occur at the start of the treatment, and the drugs are administered in combination therapy. The sequential form occurs when a patient experiences an initial severe DHSR, prompting cessation of the culprit drug and starting a second alternative therapy, all while T cells remain activated. It is not uncommon for a flare-up reaction to occur, and with prolonged treatment, a second true DHSR directed toward the alternative therapy may develop, leading to MDHS, which is frequently observed in cases of DRESS syndrome [5]. The distant or long-interval form occurs when symptoms involving the second drug appear after the resolution of the initial DHSR. The time between the first and second DHSR may range from two to 20 years [1].

Although MDHS associated with non-immediate severe reactions is widely described, MDHS in immediate reactions has been much more scarcely reported [6], and there is no proposed pathophysiological mechanism. Therefore, we report a case of immediate MDHS involving quinolones and propionic acid derivatives acquired simultaneously.

## Case presentation

We present a case of simultaneously acquired immediate hypersensitivity reactions, documented through drug challenges (DCs), to two structurally distinct drugs, which do not share pharmacological properties.

A previously healthy 30-year-old female was started on levofloxacin 500 mg and ibuprofen 600 mg for a skin infection. One hour after taking both medications, she experienced a sudden onset of mammillary and cephalic pruritus, facial and lip angioedema, hoarseness and oropharyngeal discomfort, vomiting, progressing to hypotension, and presyncope. At the emergency room (ER), anaphylaxis was not recognized, and she was treated only with antihistamines and systemic corticosteroids, being discharged 12 hours later, with improvement. Acute serum tryptase was not obtained. Approximately 12 months after the index reaction, following evaluation at our allergy and clinical immunology department, she was submitted to skin prick tests with levofloxacin (5 mg/mL) and ciprofloxacin (2 mg/mL) and intra-dermal tests with levofloxacin (0.005 mg/mL) and ciprofloxacin (0.02 mg/mL), which were negative [7]. Subsequently, she underwent a DC with levofloxacin, which was halted 25 minutes after the initial dose (125 mg) due to the occurrence of oropharyngeal discomfort, lip angioedema, dysphonia, hypotension, and bradycardia. These symptoms were treated with 0.5 mg of intramuscular epinephrine, prednisolone 40 mg, and ebastine 20 mg, with rapid resolution. Acute serum tryptase at this episode was not elevated (3.61 µg/L; basal serum tryptase = 3.74 ug/L). However, signs, symptoms, and their unfolding perfectly mimicked the index reaction. She was subsequently submitted to DC with ibuprofen, which was also positive with angioedema developing two hours after reaching 600 mg, and was treated with antihistamines and oral corticosteroids. Considering these results, IgE-mediated MDHS to levofloxacin and ibuprofen was suspected. To confirm this hypothesis, excluding COX-1-mediated hypersensitivity, a DC with acetylsalicylic acid (AAS) was performed (1 gram cumulative dose), and it was negative.

## Discussion

While the association with non-immediate severe reactions is much more common, MDHS in immediate reactions has been described, though not always highlighted [4]. A French retrospective study on perioperative anaphylaxis, with completed allergy workup, identified 10 cases (2.1%) with more than one culprit [8]. Additionally, Landry and his collaborators retrospectively reviewed a large database of patients evaluated for drug hypersensitivity in their clinic and found that 11 out of 45 cases of MDHS, confirmed through allergy workup, were IgE-mediated. Interestingly, quinolones were implicated in nine of these cases, six of which had a history of immediate reactions [9].

Recently, it has been demonstrated that quinolones can induce mast cell activation and cause degranulation through interaction with the mast cell-related G protein-coupled receptor X2 (MRGPRX2), resulting in symptoms similar to those of an IgE-mediated reaction. This makes the diagnosis of immediate hypersensitivity reactions to quinolones challenging, as there is no reliable biomarker to distinguish between the two mechanisms [10]. Although this patient presented negative skin tests to quinolones, which could suggest MRGPRX2 activation, this conclusion cannot be drawn, as there is a lack of consensus on the optimal concentrations for conducting skin tests with levofloxacin, and multiple recommendations have been proposed [10,11]. A concentration of 0.005 mg/mL was used, as previously suggested [12], but this concentration may be too low and lack sufficient sensitivity. Additionally, skin tests were conducted 12 months after the index reaction; this interval may be associated with waning IgE sensitization and a higher risk of false-negative results, given that evidence indicates up to 30% of patients with IgE-mediated penicillin allergy lose skin test positivity within one year [13].

Additionally, during DC with levofloxacin, she reacted to a small dose (125 mg), deemed insufficient to induce MRGPRX2-mediated mast cell degranulation and being more indicative of an IgE-mediated reaction [14]. She also experienced angioedema during the challenge with ibuprofen, but tolerated AAS afterward, indicating an IgE-mediated reaction to ibuprofen. Interestingly, in this case, the hypothesis of IgE-induced MDHS strongly suggested an IgE-mediated mechanism underlying ibuprofen DHS, and this rationale enabled us to confidently perform an AAS DC, excluding COX-1-mediated nonsteroidal anti-inflammatory drug (NSAID) cross-reactivity and avoiding unnecessary restrictions for the patient.

## Conclusions

MDHS is typically associated with non-immediate hypersensitivity reactions, underscoring the uniqueness of this case. Additionally, given its rarity, it is imperative for allergists to remain vigilant regarding this syndrome and to thoroughly assess all implicated drugs in ongoing allergy diagnostic workup, even if one of the drugs involved has already tested positive. Moreover, continued research is crucial for deepening our understanding of its pathophysiology and enhancing patient care approaches.
